# To Subscribers

**Published:** 1857-12

**Authors:** 


### EDITORIAL AND MISCELLANEOUS.

### To Subscribers.

It was announced at the commencement of this volume, that the publication of this Journal had been transferred to Messrs. G. P. Eddy & Co. We would now state that this arrangement has been cancelled, and the Journal will be published as before. All communications relating to the subscriptions or the business affairs of the Journal should be directed to J. G. Westmoreland, M. D., Treasurer, and all having reference to the Editorial department should be directed to the Editors.

